# In-Hospital Outcomes of Right Minithoracotomy *vs*. Periareolar Access for Minimally Invasive Video-Assisted Mitral Valve Repair

**DOI:** 10.21470/1678-9741-2020-0507

**Published:** 2022

**Authors:** Karen Amanda Soares de Oliveira, Ana Carolina dos Santos Lousa, Marcos Loiola de Souza, Tércio Campos Leão Neto, Jeffchandler Belém de Oliveira, Lucas Henrique Prado Sousa, Arlindo Rodrigues Galvão Filho, Rodrigo Oliveira Rosa Ribeiro de Souza

**Affiliations:** 1 Faculdade de Medicina, Universidade Federal de Goiás, Goiânia, Goiás, Brazil.; 2 Cardiovascular Surgery Department, Hospital Ruy Azeredo, Goiânia, Goiás, Brazil.; 3 Computer Department, Pontifícia Universidade Católica de Goiás, Goiânia, Goiás, Brazil.; 4 Cardiovascular Surgery Department, Hospital do Coração Anis Rassi, Goiânia, Goiás, Brazil.

**Keywords:** Mitral Valve, Thoracic Surgery, Thoracotomy, Cardiac Surgical Procedures, Airway Extubation, Drainage, Intensive Care Units, Length of Stay

## Abstract

**Introduction:**

In minimally invasive mitral valve repair, right minithoracotomy is the most widely performed method, providing a good view of the mitral valve. But regarding other techniques and although it offers limited visualization, the periareolar access is a less traumatic alternative. This study’s purpose is to compare in-hospital outcomes in patients who underwent video-assisted minimally invasive mitral valve repair via right minithoracotomy and periareolar access.

**Methods:**

This is a retrospective observational study including 37 patients (> 18 years old), without previous right thoracic surgery, who underwent their primary mitral valve repair, with indication for minimally invasive video-assisted approach (via right minithoracotomy or periareolar access), between January 2018 and August 2019. Patients’ medical records were consulted to collect demographics data, operative details, and in-hospital outcomes.

**Results:**

Twenty-one patients underwent right minithoracotomy, and 16 were operated via periareolar access. The mean patients’ age was 62±12 years in the right minithoracotomy group and 61±9 years in the periareolar access group (*P*=0.2). There are no significant differences in incision length, cardiopulmonary bypass time, aortic cross-clamping time, hematocrit, amount of chest tube drainage, and intensive care unit and in-hospital length of stay. Time to extubation presented significant differences between the right minithoracotomy and the periareolar access group (4.85 hours *vs*. 5.62 hours, respectively) (*P*=0.04).

**Conclusion:**

In this study, we found similar results in the two applied surgical techniques, except for the time to extubation.

**Table:** 

Abbreviations, acronyms & symbols	
CPB	= Cardiopulmonary bypass
EuroSCORE	= European System for Cardiac Operative Risk Evaluation
ICU	= Intensive care unit
LVEF	= Left ventricular ejection fraction
MIS	= Minimally invasive surgery
PA	= Periareolar access
RT	= Right minithoracotomy

## INTRODUCTION

The mitral valve repair is considered the gold standard treatment for mitral regurgitation, and it correlates with a high repair rate and low mortality^[1]^. The procedure can be performed per minimally invasive surgery (MIS), which demonstrates safety to treat a wide range of pathologies, including mitral valvopathy^[2,3]^. A recent meta-analysis showed that a minimally invasive approach to valve repair seems to provide equivalent early and long-term results to conventional median sternotomy for complex mitral valve insufficiency^[4]^. Even though median sternotomy remains a well-accepted option for mitral valve repair, minimally invasive techniques provide smaller incisions and, consequently, less operative trauma. Thus, the advantages of these are less bleeding, shorter recovery time, and more satisfactory esthetic results^[5-7]^.

Since the first successful video-assisted surgery for mitral valve repair in the 1990s, the emergence of new technologies benefited the development of different access methods^[8]^. The chosen technique must provide an adequate view of the mitral valve and allow dexterity to manipulate long surgical instruments. The right minithoracotomy (RT) satisfies these requirements; therefore, it is the most performed approach for minimally invasive mitral valve repair in most hospitals^[9]^. The periareolar access (PA), a variation of RT, offers more limited visualization compared to RT. Despite that, it is becoming more common in mitral and tricuspid valve surgeries, given that it provides a less traumatic option, showing good esthetic and sensory function results^[9-11]^.

Nevertheless, the results obtained with these two access approaches have not been compared. Consequently, an incision site choice usually depends on the surgeon’s preference and the patient’s general anatomic characteristics^[9]^. Although PA carries a potentially smaller incision, offering an esthetic advantage over RT, it is essential to investigate if these techniques showed differences regarding intraoperative management and patient’s postoperative evolution concerning esthetic and clinical parameters. This knowledge serves as a decision support tool for the cardiovascular surgeon, aiming to choose the adequate alternative for each patient.

Therefore, the purpose of this study is to compare in-hospital outcomes of patients with degenerative mitral valve regurgitation who underwent video-assisted minimally invasive mitral valve repair via RT and PA.

## METHODS

### Study Design and Patients

We performed a retrospective observational study including 37 patients with degenerative mitral valve regurgitation. The patients included were more than 18 years old and underwent their primary mitral valve repair, with an indication for minimally invasive video-assisted approach (via RT or PA), between January 2018 and August 2019. No patients were excluded from the sample. Patients’ medical records were the source of all demographics data, preoperative and intraoperative details, and in-hospital outcomes. The Research Ethics Committee approved this research of the Universidade Federal de Goiás (registration number: 3750477; CAAE number: 23020719600005078). Since all data were collected retrospectively and managed anonymously, patient informed consent was waived. This study followed the main available reporting guidelines Strengthening the Reporting of Observational Studies in Epidemiology (or STROBE)^[12]^.

### Operative Methods

Operations were carried out using general anesthesia, and patients underwent orotracheal intubation with a double-lumen endotracheal tube. Cardiopulmonary bypass (CPB) was initiated through the femoral artery and vein cannulation, arterial cannulation was performed with a femoral cannula, and venous cannulation was accomplished with percutaneous cannulation of the femoral vein.

Regarding the incisional approaches used, MIS mitral valve repair was performed through RT or PA. The choice of the incision sites was based on anatomical principles for each patient to provide ideal exposure and adequate access to the mitral valve. RT and PA have almost the same absolute contraindications (previous right hemithorax surgery and local radiotherapy), but areola diameter and absence of breast implants were appreciated when PA was considered. Therefore, PA was performed in patients with an areola diameter > 3-4 cm, and RT was performed in patients with an areola diameter ≤ 3-4 cm or patients with breast implants to avoid complications of prosthesis manipulation.

**Fig. 1:**
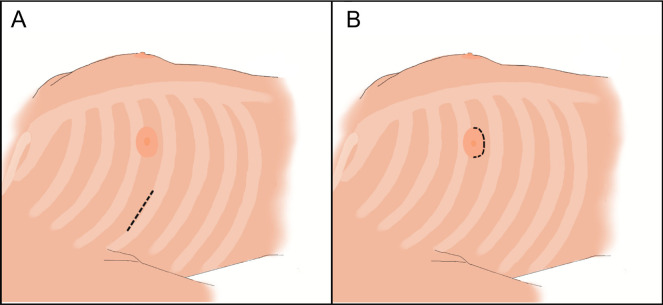
(A) Right minithoracotomy and (B) periareolar access incisions for minimally invasive mitral valve surgery.

RT was carried out through an anterolateral chest wall incision, which was performed at the fourth intercostal space in men or at the inframammary space in women. PA consists of a right lateral chest wall incision around the bottom of the nipple (Figure 1). Transthoracic aortic cross-clamping was performed, and all operations were carried out by using special instruments for minimally invasive video-assisted cardiac surgery. Mitral valve was exposed, followed by careful evaluation of the leaflets and the subvalvular apparatus, and, at last, the planning of valve reconstruction. All patients received mitral annuloplasty using semi-rigid mitral rings, and several techniques were used for the mitral valve reconstruction. The same team of surgeons performed all operations.

### Statistical Analysis

Continuous variables are expressed as mean ± standard deviations and were compared using Student’s *t*-test with Levene’s test for homogeneity of variance. Categorical variables are expressed as numbers and percentages, and Fisher’s exact tests were applied to compare the two groups’ results. We used MATLAB® Software (The MathWorks, Inc.) for statistical analysis. *P*-value < 0.05 was considered statistically significant.

## RESULTS

Among patients who underwent mitral valve repair for degenerative mitral regurgitation through a minimally invasive video-assisted access (via RT or PA), RT approach was performed in 21 (56.7%), and 16 (43.3%) underwent PA. Table 1 presents the patients’ baseline demographics and preoperative characteristics. There are no statistical differences between the RT group and the PA group for all baseline characteristics considered. All operations were completed as planned, and there were no conversions to full sternotomy or surgery for mitral valve replacement. There were no cases of in-hospital mortality in both groups.

**Table 1:** Baseline characteristics of patients who underwent MIS mitral valve repair via RT and PA.

Characteristic	RT (n=21)	PA (n=16)	*P*-value
Age (years)	62.33±12.04	61.37±9.99	0.20
Male, n (%)	18 (85.71%)	11 (68.75%)	0.21
Female, n (%)	3 (14.28%)	5 (31.25%)	
LVEF (%)	57.90±9.26	54.62±9.26	0.17
EuroSCORE II (%)	4.03±7.12	4.60±8,12	0.56
Hematocrit (%)	33.33±2.08	32.75±2.59	0.16

EuroSCORE=European system for cardiac operative risk evaluation; LVEF=left ventricular ejection fraction; MIS=minimally invasive surgery; PA=periareolar access; RT=right minithoracotomy

Table 2 summarizes intraoperative and in-hospital postoperative outcomes in each group. Time to extubation presented a statistically significant difference between the RT and PA groups (4.85±1.71 hours *vs*. 5.62±1.08 hours, respectively; *P*=0.04). There were no statistically significant differences in other intraoperative outcomes or in-hospital postoperative outcomes.

**Table 2:** Intraoperative and in-hospital postoperative outcomes of patients who underwent MIS mitral valve repair via RT and PA.

Outcome	RT (n=21)	PA (n=16)	*P*-value
Intraoperative outcomes			
Incision length (cm)	3.71±0.64	4.25±1.06	0.25
CPB time (minutes)	113.52±30.45	119.31±32.33	0.84
Aortic cross-clamping time (minutes)	81.33±18.41	82.06±21.84	0.28
Conversion to sternotomy, n (%)	0 (0%)	0 (0%)	-
In-hospital postoperative outcomes			
Time to extubation (hours)	4.85±1.71	5.62±1.08	0.04
Chest tube drainage (ml/24 hours)	323.81±139.3	309.37±162.5	0.43
Transfusion of red blood, n (%)	0 (0%)	0 (0%)	-
ICU length of stay (hours)	34±16.46	29.62±16.6	0.54
Total length of stay (days)	4.09±0.53	4.25±0.44	0.79
Hematocrit (%)	33.33±2.08	32.75±2.59	0.16
In-hospital death, n (%)	0 (0%)	0 (0%)	-

CPB=cardiopulmonary bypass; ICU=intensive care unit; MIS=minimally invasive surgery; PA=periareolar access; RT=right minithoracotomy

## DISCUSSION

Over the last decades, the creation of new technologies and access methods to the thoracic cavity turned the mitral valve repair less invasive, shortening the incision length and lessening the patient’s postoperative recovery time. Our study compared two minimally invasive techniques for video-assisted mitral valve repair (RT and PA), which showed similar results. Time to extubation was the only variable with a statistically significant difference between the RT and PA groups.

The equivalence between these techniques’ results is not surprising, considering that the two approaches involve an incision at the fourth intercostal space. Nevertheless, each method presents its particularities. RT provides a more direct view of the valve at the expense of an increased distance from the intracardiac structures. In contrast, PA consists of a more medial and delicate incision, offering closer access to the mitral apparatus, with less operative trauma since it does not require the excessive spreading of the ribs^[10,13]^. However, RT remains the most well-accepted approach for MIS mitral valve repair worldwide, presenting low morbimortality in the short and long terms^[13-16]^.

Furthermore, PA is an alternative for RT in patients with favorable anatomy, and we performed it in 16 (43.3%) patients of this study (11 [73.33%] males). However, Poffo et al.^[11,17]^ (2018) demonstrated the success of the video-assisted PA even in a sample composed mostly of women. Their study concluded that the presence of large breasts makes it difficult to access the intercostal space and the surgical view per RT, turning PA into an excellent alternative. Although the incision’s mean length has been higher in the PA group, this incision provides a discrete scar since it is placed in the nipple-areolar complex transition. Accordingly, patients who underwent PA refer to high satisfaction levels through the patient scar assessment scale and dermatology life quality indexes^[10,12]^.

Although MIS presents a longer operative time compared to the open approach, it does not implicate significant postoperative complications^[18]^. An analysis conducted by Percy et al.^[14]^ (2020) showed that patients who underwent mitral valve repair via hemisternotomy had shorter CPB and aortic cross-clamping times than patients who underwent RT, an expected result given the higher correspondence between hemisternotomy and the conventional approach^[14]^. In our study, CPB and aortic cross-clamping times were higher in the PA group but without significant difference. Satisfactorily, CPB and aortic cross-clamping times in mitral valve MIS have shown a considerable reduction as the surgical team gains experience, which relates to better surgical results^[19,20]^.

The mean time to extubation was significantly longer in patients who underwent MIS mitral valve repair via PA (*P*=0.04), and it may reflect in the longer operative time of this group. Moreover, the anesthetic plan factors also play an essential role in determining extubation time^[21]^. Still, the volume drained from the chest in 24 hours had a lower mean value in the PA group (*P*=0.43). The minor operative trauma described for MIS resulted in less fluid drained in the postoperative period and less need for blood transfusions^[6,7,21]^. Therefore, fully thoracoscopic procedures for mitral valve surgery, including fully thoracoscopic PA, have gained popularity as even less invasive approaches^[18,22]^.

The average length of stay in the intensive care unit (ICU) was slightly higher in the RT group, which is not statistically significant (0.54) and, in general, it was similar to that described in other studies^[14-16]^. In a study conducted at the Massachusetts General Hospital, the average length of stay in the ICU of patients who underwent MIS mitral valve repair was only 24 hours^[6]^. The reduction in the total length of hospital stay demonstrates a fast recovery of the patient, and our analysis found an average of four days until hospital discharge. Furthermore, the in-hospital mortality of 0% reaffirms the safety of RT and PA. Similarly, Percy et al.^[14]^ (2020) described mortality of 0% and 0.7% in 30 days for RT and hemisternotomy for mitral valve repair, respectively.

Despite the two techniques studied presented similar results, a discrete access site and a better cosmetic effect are among the PA advantages. Furthermore, the absence of ribs excessive spreading is responsible for reducing postoperative pain^[23]^. Other scientific evidence also demonstrated satisfactory results of other minimally invasive methods of access^[24,25]^. However, the adoption of these techniques still requires overcoming a long learning curve^[18]^. Furthermore, with the performance of increasingly smaller incisions in video-assisted surgeries, adequate video assistance is essential, requiring adaptation of the operating room. Therefore, cardiovascular surgeons must acquire the necessary competence to apply these innovations, expanding their field of action to offer the best treatment to all patients.

### Limitations

The limitations of our study are the limited number of operated patients and the learning curve of the team responsible for the procedures, which cannot be generalized for all circumstances.

## CONCLUSION

In this study, we found similar results in the two surgical techniques applied, except for the time to extubation, which was lower in patients who underwent MIS mitral valve repair via RT. Both incisional approaches were feasible and demonstrated optimal in-hospital results. Further studies are needed to compare the results obtained with RT and PA in larger samples size and evaluate long-term outcomes achieved with each incisional approach.

**Table:** 

Authors' roles & responsibilities	
KASO	Substantial contributions to the conception and design of the work; and the analysis or interpretation of data for the work; drafting the work or revising it critically for important intellectual content; final approval of the version to be published
ACSL	Drafting the work or revising it critically for important intellectual content; final approval of the version to be published
MLS	Substantial contributions to the analysis or interpretation of data for the work; final approval of the version to be published
TCLN	Substantial contributions to the acquisition of data for the work; final approval of the version to be published
JBO	Substantial contributions to the acquisition of data for the work; final approval of the version to be published
LHPS	Substantial contributions to the acquisition of data for the work; final approval of the version to be published
ARGF	Substantial contributions to the analysis and interpretation of data for the work; final approval of the version to be published.
RORRS	Substantial contributions to the acquisition of data for the work; drafting the work or revising it critically for important intellectual content; final approval of the version to be published
